# Upgrading Solid Digestate from Anaerobic Digestion of Agricultural Waste as Performance Enhancer for Starch-Based Mulching Biofilm

**DOI:** 10.3390/molecules26040832

**Published:** 2021-02-05

**Authors:** Nan Zhao, Huawei Mou, Yuguang Zhou, Xinxin Ju, Shoujun Yang, Shan Liu, Renjie Dong

**Affiliations:** 1 Bioenergy and Environment Science & Technology Laboratory, College of Engineering, China Agricultural University, Beijing 100083, China; nan.zhaoca@outlook.com (N.Z.); mouhuawei@163.com (H.M.); rjdong@cau.edu.cn (R.D.); 2 Key Laboratory of Clean Production and Utilization of Renewable Energy, Ministry of Agriculture and Rural Affairs, Beijing 100083, China; 3 National Center for International Research of BioEnergy Science and Technology, Ministry of Science and Technology, Beijing 100083, China; 4 Prataculture Machinery and Equipment Research Center, College of Engineering, China Agricultural University, Beijing 100083, China; zhouyg@cau.edu.cn; 5 State R&D Center for Efficient Production and Comprehensive Utilization of Biobased Gaseous Fuels, National Energy Administration, Beijing 100083, China; 6 National Energy R&D Center for Biomass, China Agricultural University, Beijing 100193, China; 7 Shandong Sino-March Environmental Technology Co., Ltd., Yantai 264006, China; jxx0617@126.com; 8 Yantai Institute, China Agricultural University, Yantai 264670, China; sjyang-2008@163.com

**Keywords:** bioplastic, mulching film, mechanical performance, greenhouse gas emission, waste recycle

## Abstract

Developing a green and sustainable method to upgrade biogas wastes into high value-added products is attracting more and more public attention. The application of solid residues as a performance enhancer in the manufacture of biofilms is a prospective way to replace conventional plastic based on fossil fuel. In this work, solid digestates from the anaerobic digestion of agricultural wastes, such as straw, cattle and chicken manures, were pretreated by an ultrasonic thermo-alkaline treatment to remove the nonfunctional compositions and then incorporated in plasticized starch paste to prepare mulching biofilms by the solution casting method. The results indicated that solid digestate particles dispersed homogenously in the starch matrix and gradually aggregated under the action of a hydrogen bond, leading to a transformation of the composites to a high crystalline structure. Consequently, the composite biofilm showed a higher tensile strength, elastic modulus, glass transition temperature and degradation temperature compared to the pure starch-based film. The light, water and GHG (greenhouse gas) barrier properties of the biofilm were also reinforced by the addition of solid digestates, performing well in sustaining the soil quality and minimizing N_2_O or CH_4_ emissions. As such, recycling solid digestates into a biodegradable plastic substitute not only creates a new business opportunity by producing high-performance biofilms but also reduces the environmental risk caused by biogas waste and plastics pollution.

## 1. Introduction

The plastic film technology is widely employed in social development due to its economic suitability and easy processing [1,2]. A plastic mulching film is often used to protect agricultural soil, aid establishment and accelerate the growth of crops in the seeding stage and reduce GHG (greenhouse gas) emissions from farmlands [3,4]. The successful practice has been used in agricultural fields in the past decades, and crops planted in mulching surroundings can achieve high quality and productivity. However, the popularity of plastic film also causes a high risk of white pollution, because there are many health-threatening substances emitted from plastic production, and it is also difficult to recycle in soil [5,6]. As a result of the accumulation of plastic residues in farmlands, the root developments of crops will be seriously destroyed [7]. Normally, waste plastic is usually disposed by the landfill tipping method but with high labor and energy costs, and, subsequently, will be decomposed in soil for 200 or more years [8]. Moreover, conventional plastic generally originates from finite fossil fuel resources, and its overuse has caused an energy crisis [9]. Therefore, it is necessary to promote a degradable film based on renewable resources to protect the agroecological environment and compensate the consumption of fossil energy [10].

A starch-based biofilm is a sustainable alternative for farmers to replace plastic films in farmlands, because this biofilm is biodegradable, and starch resources are abundant and have low costs [11]. The traditional starch-only biofilm is poor-performing because of poor mechanical properties and hydrophilic characteristics, and these disadvantages can be overcome by blending other additives [12]. Previous research has confirmed that cellulose can increase the tensile strength and Young’s modulus of biofilm as a reinforcement in a polymeric system [13,14]. The resource of cellulose is rich, which can be easily achieved from nature. However, there is also a limited feature when using cellulose as a performance enhancer, i.e., cellulose is always surrounded by non-decomposed lignin and is difficult to apply in the manufacturing of biofilms directly without any pretreatments [15]. Additionally, the current pretreatment methods, such as separation and extraction, consume high energy and money.

China has a long history of using anaerobic digestion technology to recycle agricultural waste and produce biogas since the 1970s [16]. In 2014, over 41.5 million household digesters and 99,957 biogas plants were in operation, producing 15.8 billion m^3^ of biogas, equal to 18.68 million tons of standard coal [17,18]. However, the rapid promotion of biogas also produced large amounts of residues, causing serious threats to the environment without inappropriate post-treatments [19]. Solid residues (or digestates) from straw and ruminant manures are rich in cellulose, widely reused as poultry litter, composting, hydrothermal carbonization, solid fuel and fertilizer for direct application to soil, but few studies have focused on recycling it in biofilm fabrication [20]. During the anaerobic digestion of a lignocellulosic biomass, most of lignin and hemicellulose are gradually degraded to small fragments; therefore, cellulose is released from the cover of lignin, as well as the removal of the amorphous region of cellulose [16]. A solid digestate can therefore be regarded as an excellent additive to improve the performance of the biofilm. It is worth noting that a solid digestate also contains some nonfunctional impurities, such as humic acid, which will weaken the intensity of a composite biofilm without pretreatment [18].

The aim of this work was to prepare a novel starch-based biofilm with the additions of solid digestates from straw and cattle and chicken manures by using the solution casting technique. For further studies and industrialization, the effects of the additive content of solid digestate on the performances of a composite biofilm were also investigated. This study can provide useful information for recycling biogas waste as a performance enhancer to produce degradable mulching film so as to replace plastic, which will contribute greatly to minimizing plastic use in farmlands.

## 2. Materials and Methods

### 2.1. Materials

Corn starch was purchased from Hebei Zhangjiakou Yujing Food Co., Ltd. (Hebei, China). Solid digestates from the anaerobic digestion (mesophilic condition) of straw and cattle and chicken manures were supplied from Doudian Biogas Station in Fangshan District in Beijing, China. Glycerol (analytical grade) and xylitol (analytical grade) were purchased from Beijing Chemical Reagent Ltd. (Beijing, China) and Tianjin Jinguigu Science & Technology Development Co., Ltd. (Tianjin, China). All other chemicals used in this study were analytical grade.

### 2.2. Composition Analysis

The contents of cellulose, hemicellulose and lignin in solid digestates were quantified by using two-step acid hydrolysis according to the National Renewable Energy Laboratory method [21]. Ash content was determined by a muffle furnace at 550 °C according to the incineration method [22]. Humic acid content was measured by examining the visible spectrum of humic acid extracted in the alkaline solution according to the spectrophotometric method [23].

### 2.3. Pretreatment

Solid digestates from the biogas plant were washed repeatedly to remove water-soluble impurities and dried at 60 °C for 24 h in a drying oven (Yiheng Scientific Laboratory Instrument Co., Ltd., Shanghai, China). The dried samples were mixed with 2% sodium hydroxide solution under the action of ultrasonic (60 °C) for 30 min in an ultrasonic cleaner (KQ5200DE, Changfeng Instrument and Meter Co., Ltd., Beijing, China) to remove humic acid and degrade hemicellulose, lignin and ash [24,25]. Precipitates after filtration were washed and dried for further use.

### 2.4. Biofilm Preparation

**Figure 1:**
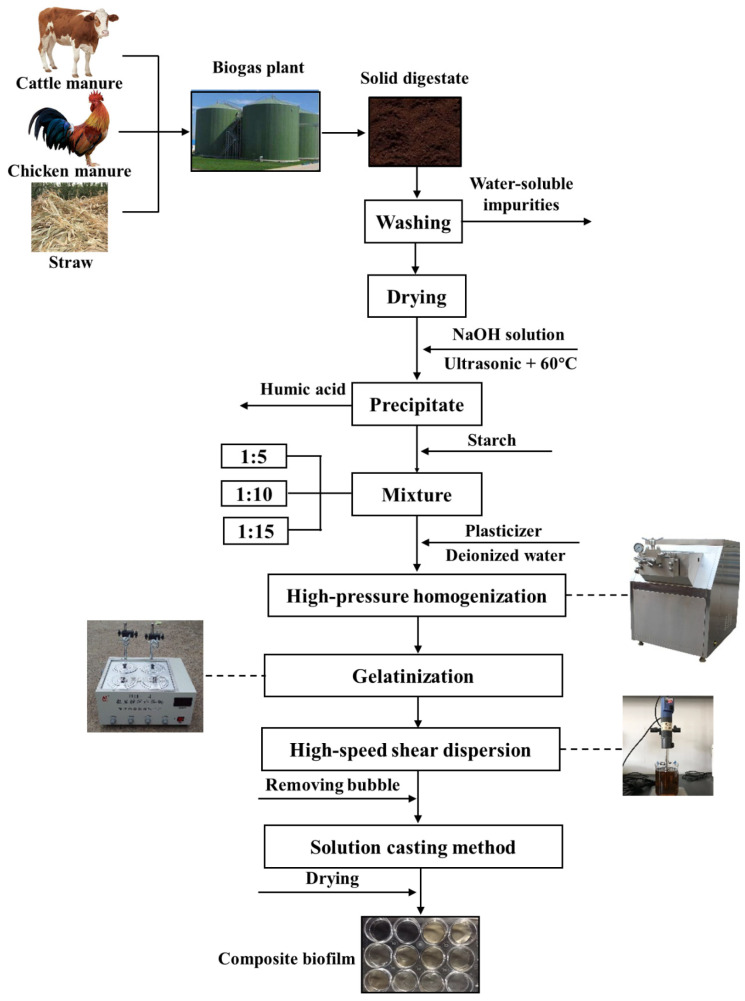
Preparation protocols of the biofilm composed with starch and the solid digestates.

Preparation protocols of the composite biofilm are shown in Figure 1. Pretreated solid digestates were mixed with starch by the ratios of 1:5, 1:10 and 1:15. The mixtures of starch and solid digestate were then mixed with plasticizers (composed with glycerol and xylitol by a ratio of 1:1) by a ratio of 7:3 and dissolved in deionized water to configure 10% (*w*/*v*) mixing solution. High-pressure homogenizer (AH100D, ATS Engineering Inc., Shanghai, China) was used to homogenize this solution three times, and the homogenized solution was heated in a 99 °C water bath (HHS-4S, Yulong Instrument Co., Ltd., Beijing, China) with a constant stirring of 300 r/min by an electronic stirrer (OS20-Pro, Dragon Laboratory Instruments Co., Ltd., Beijing, China) for 1 h to promote starch gelatinization. Evaporation was minimized by sealing the opening of the container [26]. 

The fully gelatinized solution was retired and sheared by using a high-speed disperser (T25, Truelab Lab-sci Co., Ltd., Shanghai, China) for 10 min at 6000 r/min, ultrasonically vibrated for 30 min at 60 °C and placed in a vacuum oven (DZ-3, Taisite Instrument Co. Ltd., Tianjin, China) at 45 °C for 10 min to remove air bubbles inside the solution. Five milliliters of solution were poured and spread uniformly onto a plastic dish (9 cm in diameter) and dried at 45 °C for 5 h (solution casting method). Biofilms were peeled carefully after conditioning in a constant temperature and humidity incubator (JYH-152, Jiayu Scientific Instrument Co., Ltd., Shanghai, China) at 20 °C and 43% relative humidity for 24 h to reach the equilibrium moisture content.

Film thickness was measured by an electronic digital caliper (PRO-MAX, Fred V. Fowler Co. Inc., Auburndale, MA, USA) at 5 random positions, and the moisture content was determined by a halogen moisture balance (HB43-S, Mettler-Toledo Instruments (Shanghai) Co., Ltd., Shanghai, China). As shown in Table 1, the thickness and moisture contents of all biofilms had no significant differences at the 95% confidence level. This means that the influences of the thickness and moisture content on the biofilm characteristics can be ignored in this study.

### 2.5. Morphology

The morphology of the biofilm (surface and cross-section) was visualized by scanning electron microscopy (JSM-6700F, JEOL Ltd., Akishima, Japan). Biofilm was cut, fixed on a stub with double-sided adhesive tape and coated with a thin layer of gold. Each biofilm was examined at 500× magnification for the surface and 2000× magnification for the cross-section, with an accelerating voltage of 10 kV.

### 2.6. Crystallinity

Biofilm was crushed and shifted by an 80-mesh sieve. The diffraction pattern of the biofilm powder was determined using an X-ray diffractometer (XD-2, Purkinje General Instrument Co., Ltd., Beijing, China) with a Cu kα radiation at a scanning angular range from 5° to 80°. The sampling interval was 0.02°, and the scanning rate was 4°/min. The voltage and current were set as 36 kV and 20 mA. The relative crystallinity was calculated by using Equation (1) [27].

(1) $$X_{C}=\frac{I_{C}+I_{A}}{I_{C}}$$

### 2.7. Fourier-Transform Infrared Spectroscopy (FTIR)

FTIR characterization analysis of the biofilm was performed on a Fourier-transform infrared spectrometer (Affinity-1S, Shimadzu Co. Ltd., Kyoto, Japan) at a scanning range from 4000 to 400 cm^−1^ [28]. FTIR spectra were achieved in a transmittance mode with a 4 cm^−1^ resolution at an accumulation of 100 scans.

### 2.8. Mechanical Property

The main mechanical properties, including strength, elongation at break and elastic modulus, were evaluated by a dynamic mechanical analyzer (Q800, TA Instruments Co. Ltd., Newcastle, DE, USA) in tensile mode [29]. Biofilm was cut into a 30 × 10 mm rectangular strip, clamped and applied by a 0.01 N preload to avoid buckling. Then, the strip was loaded with changing stress at a rate of 5 MPa/s until a fracture occurred. The working temperature and frequency were set as 20 °C and 1 Hz.

### 2.9. Thermal Stability

The thermal stability was analyzed by a differential scanning calorimeter (DSC-Q10, TA Instruments Co. Ltd., Newcastle, DE, USA) [27]. Biofilm was crushed, and about 3.5-mg powder was sealed in an aluminum crucible with an empty one as the control. Biofilm powders were heated from 20 °C to 300 °C at a heating rate of 10 °C/min. Glass transition temperature and melting temperature were determined from DSC thermographs.

### 2.10. Transparency

Transparency measurements were carried out by an automatic microplate spectrophotometer (SpectraMax M2^e^, Molecular Devices Instrument Co., Ltd., Shanghai, China) to determine the opacity of the biofilm. Biofilm was cut into a circular shape with a diameter of 20 mm and placed in a 12-well cell culture cluster. The absorbance spectrum was recorded at a range from 300 to 780 nm, with a spectral bandwidth of 2 nm [30]. The absorbance was then converted into transmittance using Equation (2).

(2) $$A_{b}=2-\log(100\times T_{r})$$

Light transmittance of the biofilm was determined in the UV-B region (315 mm), UV-A region (380 nm) and visible region (700 nm), respectively, in this study.

### 2.11. Water Vapor Permeability

A fifty-milliliter beaker with a 50.27 cm^2^ circular opening was sealed by the biofilm and stored in a desiccator at 20 °C. Anhydrous calcium chloride (relative humidity (RH) = 0%) was placed inside the beaker, and a saturated potassium carbonate solution (RH = 43%) was poured into the desiccator to maintain the RH gradient across the biofilm [31]. The weight of the beaker was measured every 12 h until the weight change was less than 0.01 g. The water vapor permeability (*WVP*) was calculated by the following equations [32]:

(3) $$WVTR=\frac{1}{A}(\frac{M}{t})$$

(4) $$WVP=\frac{WVTR\times T}{P\times (R_{1}-R_{2})}$$

where *P* is set as 2.3388 × 10^3^ Pa (20 °C) in this study, and *R_1_* and *R_2_* are expressed in fractions.

### 2.12. Gas Transmission Rate

The N_2_O and CH_4_ transmission permeabilities were determined by a gas permeability tester (N500Z, GBPI Co., Ltd., Guangzhou, China). Biofilm was cut into a circular shape with a diameter of 4 cm and placed in a closed chamber with two cells on both sides of the biofilm. Working temperature and relative humidity were set as 20 °C and 43%. N_2_O or CH_4_ was supplied with an inlet pressure of 2 kPa from one cell, and the other was vacuumed. The concentration of gas was measured using a multiple gas analyzer (LGR 907-0011, Yiwin Instrument and Equipment Co., Ltd., Shanghai, China).

### 2.13. Statistical Analysis

Each test was repeated at least three times, and the values were determined as the mean ± standard deviation. Duncan’s test from SPSS 20.0 software (SPSS Inc., Chicago, IL, USA) was used to check the significance of all results when necessary at a 95% confidence level. X-ray diffraction results were smoothed by using the FFT filter of Origin Pro 8.0 software (OriginLab Co., Northampton, MA, USA).

## 3. Results and Discussion

### 3.1. Pretreatment of Solid Digestates

As shown in Table 2, hemicellulose, lignin, ash and humic acid in the solid digestates were degraded by thermo-alkaline with an ultrasonic-assisted pretreatment while the relative content of the cellulose increased. The highest removal efficiency of humic acid was 58.85% for the solid digestate of cattle manure, the highest removal efficiencies of lignin and ash were 43.19% and 44.11% for straw and the highest removal efficiency of hemicellulose was 36.66% for chicken manure. Moreover, the growth rate of cellulose in the solid digestate of cattle manure (63.88%) was highest, followed by straw (46.94%) and chicken manure (5.10%). This indicated that the pretreatment method was more suitable for solid digestates of straw and cattle manure than chicken manure, because there were more complex compositions in the solid digestate of chicken manure, such as crude protein, which inhibited the removal effect of the pretreatment method [33].

### 3.2. Morphology

**Figure 2:**
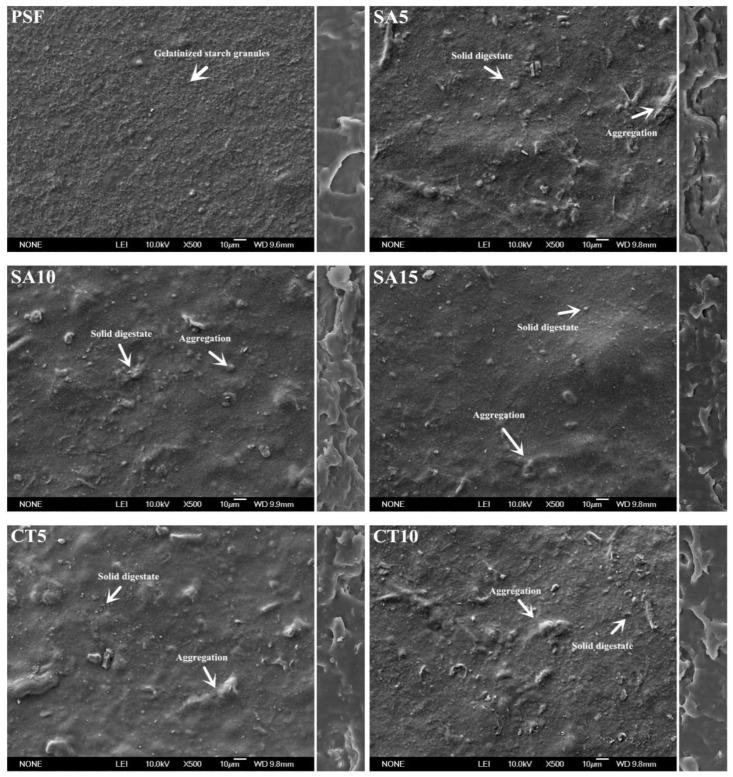

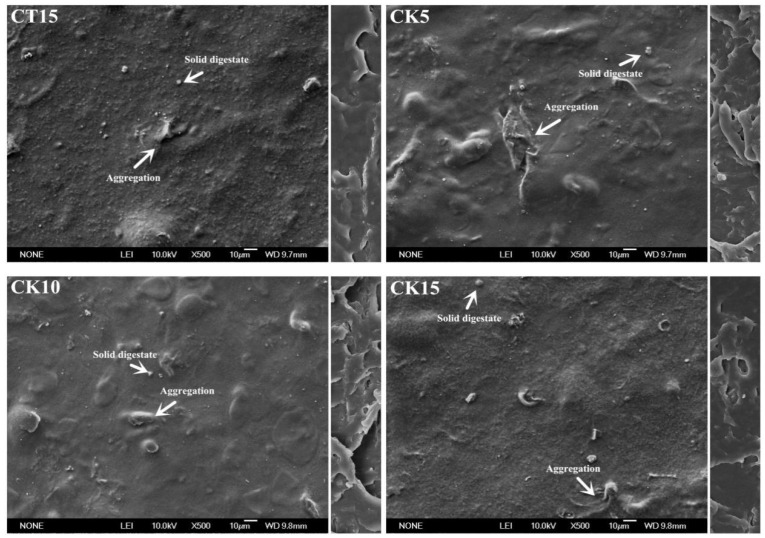
SEM of the surface (**left**) and cross-section (**right**) of the composite biofilms (SA5, SA10, SA15, CT5, CT10, CT15, CK5, CK10 and CK15) and PSF.

The morphology structures of the surface and cross-sections of the biofilms are shown in Figure 2. The SEM images of PSF (pure starch film) presented a uniform and compact surface microstructure with a dense arrangement of gelatinized starch granules. There was no protuberance observed in PSF, because the presence of the plasticizer disrupted completely the intermolecular and intramolecular hydrogen bonds of the native corn starch during biofilm formation [31,34]. Comparatively, the surfaces of the biofilms with the addition of a solid digestate showed a relatively rough microstructure with a homogeneous dispersion of solid digestate particles in the starchy matrix. There were also some aggregations detected on the composite biofilm surface, leading to a direct transformation of the biofilm structure to a more rigid state than PSF [13]. As compared with the SEM morphology of the solid digestate without any pretreatment, the ultrasonic thermo-alkaline method decreased its particle size significantly in this work [35]. Previous research has indicated that the small particle size of solid digestates can make them easy to incorporate into a bio-based matrix [36].

There was a clear scaly structure shown in the cross-section of all biofilms. This is a typical multilaminar structure, the advantage of which can generally help improve the performance of a biofilm. As shown in the cross-section of the composite biofilm, solid digestate particles were well-incrusted in the scaly structure under the action of strong adhesion between starch and cellulose in the solid digestates with the addition of plasticizers, directly stabilizing the whole biofilm structure [26].

### 3.3. Crystallinity

**Figure 3:**
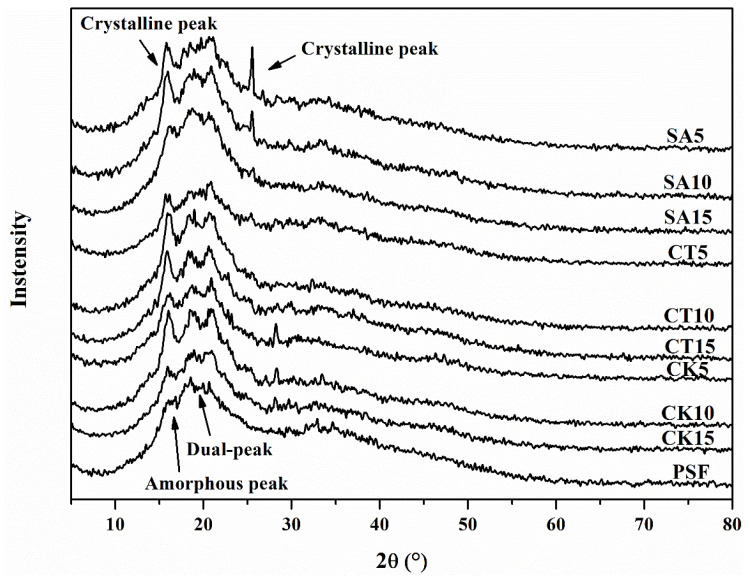
X-ray diffraction patterns of SA5, SA10, SA15, CT5, CT10, CT15, CK5, CK10, CK15 and PSF.

As shown in the X-ray diffraction (XRD) pattern of PSF, there was an overlapping peak at 16.08° (2*θ* value) and two dual-peaks at 18.24° and 19.60° (Figure 3), similar with corn starch (A-type structure). However, the overlapping peak of PSF was an amorphous halo peak, which was different from net starch because of the addition of a plasticizer, which disorganized the starch granules [34]. Compared with PSF, the composite biofilms had a new sharp peak at 15.44° for the solid digestates of straw and cattle and chicken manures, the intensity of which increased with the increase of the addition of the solid digestates. Additionally, there were also obvious peaks at 25.42° for the straw digestate and 28.16° for the chicken digestate. These new peaks were related to the polymorphs structures of cellulose I and II [37]. Moreover, there was also a transformation of the peak at 16.08° from an amorphous form to a crystalline one after the incorporation of a solid digestate in PSF.

The relative crystalline degrees of all the biofilms are listed in Table 3. The crystallinity of the composite biofilm was significantly higher than that of PSF due to the high crystalline nature of cellulose in the solid digestates [38]. As reported, anaerobic digestion can efficiently remove the amorphous region of cellulose until only the crystalline one remains [39]. This means that the addition of a solid digestate increased the crystalline degree of the biofilm more significantly than natural cellulose. The highest crystalline degree was found in the biofilm enhanced by the solid digestate of straw, followed by the cattle and chicken manures.

### 3.4. FTIR Analysis

**Figure 4:**
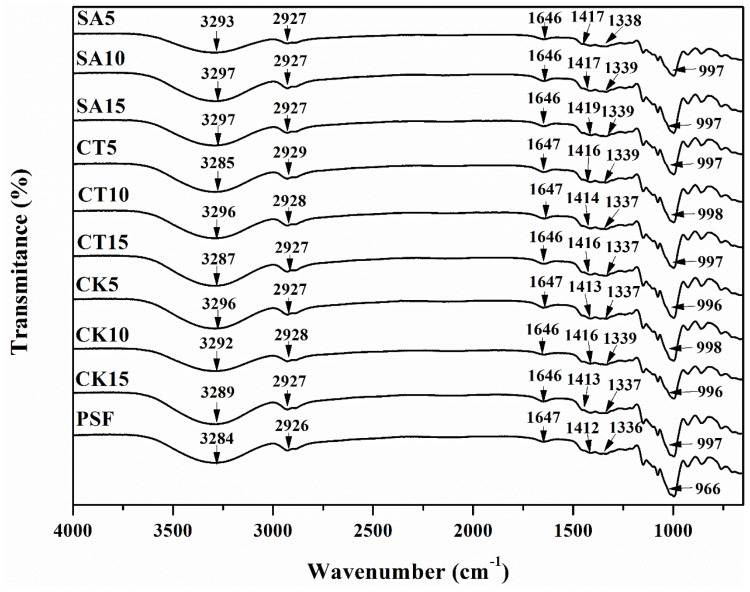
Fourier-transform infrared (FTIR) spectra of the composite films (SA5, SA10, SA15, CT5, CT10, CT15, CK5, CK10 and CK15) and PSF.

The physical blends and chemical interactions of the composite materials were, in general, affected by changes in the characteristics of the spectral bands [36]. The FTIR spectra of the composite biofilm was similar with that of PSF (Figure 4) because of the chemical similarity between starch and cellulose in the solid digestates [40]. As shown in PSF, the absorption bands at 996, 1336, 1412, 1647, 2926 and 3284 cm^−1^ were related to the stretching vibrations of the -C-O groups and the asymmetric modes of the C=O groups, plasticizer, bound water, the -CH groups and the -OH groups [41]. There were also some weak bands observed at low wavenumbers, which were related to the skeletal vibrations of the glucose rings [38]. All the absorption bands of the biofilm were shifted to relatively high wavenumbers (except for 1647 cm^−1^) after the addition of a solid digestate because of the formation of hydrogen bonding between the hydroxyl groups in starch and carboxyl groups in the solid digestates [42]. Moreover, the band intensity of the composite film was higher than that of PSF. The changes in the FTIR spectra indicated an obvious improvement in the compatibility and structural stability of the biofilm enhanced by the solid digestates.

### 3.5. Mechanical Properties

Table 3 showed that the addition of a solid digestate improved the strength and elastic modulus of the biofilm but at the expense of elongation. The mechanical strength of the composite film in this work was nearly twice higher than that of the biofilm based on thermoplastic starch (only 3.3 MPa) [43] and even greater than the biofilm composite of starch and cellulose nanofiber (6.9 MPa) [44]. The increase in rigidity was attributed to the similarity of the polysaccharide structures between cellulose and starch [40], while the decrease in plasticity was because the rigid nature of cellulose in the solid digestates increased the starch viscosity and inhibited the motion of the polymer matrix [45]. Moreover, the addition of a solid digestate on the starchy matrix stabilized the skeleton structure of the biofilm and reduced the molecular mobility, also explaining the high elasticity and low plasticity in the composite biofilm [46]. In this study, micro-interactions between the starch and solid digestate and crystalline degree correlated positively with the mechanical performances of the biofilm. 

The composite biofilms enhanced by a solid digestate of straw or cattle manure exhibited higher strength and elastic modulus than chicken manure, because the solid digestates of straw and cattle manure contained higher cellulose contents, which improved the elastic mechanical performance of the biofilm more significantly. Lignin is often considered as a highly reactive thermoplastic polymer, interacting with glycerol, xylitol and starch and acting as an interfacial compatibilizer between cellulose and starch [46]. This means that lignin can also improve the mechanical performances of biofilms to a certain extent, although the content of which is limited in a solid digestate. Additional contents of the solid digestates in the composite biofilms enhanced the mechanical performances more obviously, including increasing the elastic modulus and decreasing the elongation. However, there was also a slight reduction in the strength of the composite biofilms when adding more solid digestates, such as solid digestates of straw and chicken manure.

### 3.6. Thermal Stability

**Figure 5:**
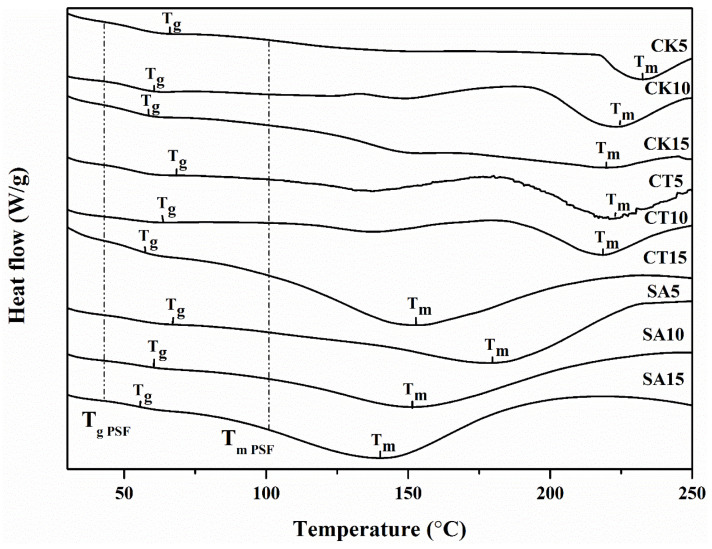
Differential scanning calorimeter (DSC) curves of the composite biofilms (SA5, SA10, SA15, CT5, CT10, CT15, CK5, CK10 and CK15) and PSF. *T_g_* and *T_m_*: glass transition temperature and degradation temperature, respectively.

The thermal characteristics of PSF and the composite biofilms measured by DSC are depicted in Table 3 and Figure 5. There were two clear endothermic transitions in the DSC curve of each biofilm, where the first one was a slight step change associated with the phase transition temperature, and the second one was a sharp galley over a broader temperature range related to the degradation temperature [47]. The values of the glass transition temperature (*T_g_*) and degradation temperature (*T_m_*) of the composite biofilms were higher than those of PSF, indicating an excellent improvement in the thermal stability of the biofilms caused by the incorporation of solid digestates in the starch matrix. This was because the addition of a solid digestate increased the regularity, compaction and crystalline degree of the biofilm, which correlated positively with the thermal performance of the biofilm. Ash in a solid digestate is considered a high-temperature-resistant material, which can improve the thermal performance of a biofilm by complicating the association, aggregation and helix structure of the starch–cellulose matrix [48]. The highest *T_g_* value was observed in the composite biofilm enhanced by the solid digestate of straw, followed by cattle manure and chicken manure, because the solid digestate of straw contained more ash. Moreover, the highest *T_m_* value was found in the composite biofilm enhanced by the solid digestate of cattle manure, followed by chicken manure and straw.

### 3.7. Transparency

The detailed transmittances at the UV-B, UV-A and Visible regions are presented in Table 3, and the results showed a lower transparency in the composite biofilm than PSF. A solid digestate is a good light-absorbing agent, the fine particles of which can be incorporated well into a starch matrix and inhibit light transmitted through a biofilm [49]. This indicated that the addition of solid digestates improved the film-barrier property against the UV and visible light radiation of the biofilms, which was also further enhanced with more additions of the solid digestates. Moreover, the light barriers of the composite biofilms were more significant for UV light than visible light. The best light barrier performance of a biofilm was achieved after adding the solid digestate of straw, followed by cattle and chicken manures, because of the relatively less impurity composition and highest cellulose content in straw, which contributed to improving the light barrier of the biofilm [9]. There are lots of advantages when using a mulching film with excellent light barrier performance in farmlands, such as inhibiting weed growth, keeping the soil temperature stable and increasing the crop yields.

### 3.8. WVP and GTR

As shown in Table 3, the composite biofilm had a lower WVP value compared to PSF. This meant that the addition of a solid digestate improved the water barrier property of the biofilm because of the strong interfacial adhesion between starch and cellulose in a solid digestate directly reduced water the vapor permeability of the biofilms [50]. The hydrogen bond formed in a mixture of starch and cellulose limited the formation of a void on the biofilm surfaces where water molecules could not pass [49]. Moreover, the water barrier performance of the composite biofilm enhanced by the solid digestate of cattle manure was better than the other composite biofilms. There are some benefits of applying mulching films with excellent water barrier performances to agricultural crops, including conserving the soil moisture and improving the crops’ growth.

The GTRs of N_2_O and CH_4_ were also reduced significantly by incorporating a solid digestate in a biofilm as an enhancer, because the solid digestate increases the density and crystalline degree of a biofilm, which relates negatively to the permeability of GHG. Moreover, the effect of a tortuous path caused by the addition of a solid digestate in a starch matrix lengthened the gas diffusive process when migrated through a biofilm, which also reduced the GTR of GHG [9]. N_2_O and CH_4_ are, in general, the main GHGs emitted from farmlands due to the application of nitrogen fertilizers. The decrease in the permeability of N_2_O and CH_4_ from farmlands by using mulching films can reduce GHG emissions to atmosphere, so as to lessen the risk of global warming.

## 4. Conclusions

The solid digestates of straw and cattle and chicken manures were pretreated by thermo-alkaline with the ultrasonic-assisted method to remove the nonfunctional compositions and then recycled as a performance enhancer in a starch-based biofilm. The addition of solid digestates stabilized the structure of the starch matrix and increased the crystalline degree of the biofilm, therefore improving the tensile strength and elastic modulus of the biofilm but at the expense of elongation. The values of *T_g_* and *T_m_* of the composite biofilm were much higher than PSF, exhibiting an excellent thermal stability. There was also a great potential of improving the light, water and gas barrier performances when incorporating a solid digestate into biofilm manufacturing. Particularly a composite biofilm with low GTRs of N_2_O and CH_4_ can reduce the emission of GHG from farmlands when using it as the mulching film. Moreover, the different additional contents and types of solid digestates also affect the biofilm performances. Upgrading solid digestates as performance enhancers for starch-based biofilms can provide a high-value use of biogas waste, and composite biofilms will become a promising alternative for mulching materials used on farmlands to improve the soil quality and protect the agricultural ecosystem.

## Figures and Tables

**Table 1:** The code and thickness of the mulching films used in this study.

No.	Code	Solid Digestate	Solid Digestate/Starch	Thickness (mm)	Moisture Content (%)
1	SA5	Straw	1:5	0.053 ± 0.005 ^a^	13.45 ± 0.15 ^a^
2	SA10	Straw	1:10	0.052 ± 0.004 ^a^	13.59 ± 0.21 ^a^
3	SA15	Straw	1:15	0.049 ± 0.007 ^a^	13.43 ± 0.20 ^a^
4	CT5	Cattle manure	1:5	0.049 ± 0.007 ^a^	13.54 ± 0.19 ^a^
5	CT10	Cattle manure	1:10	0.049 ± 0.004 ^a^	13.44 ± 0.11 ^a^
6	CT15	Cattle manure	1:15	0.054 ± 0.005 ^a^	13.33 ± 0.45 ^a^
7	CK5	Chicken manure	1:5	0.056 ± 0.009 ^a^	13.31 ± 0.11 ^a^
8	CK10	Chicken manure	1:10	0.052 ± 0.004 ^a^	13.53 ± 0.29 ^a^
9	CK15	Chicken manure	1:15	0.055 ± 0.005 ^a^	13.33 ± 0.30 ^a^
10	PSF	Pure starch film	/	0.053 ± 0.002 ^a^	13.74 ± 0.19 ^a^

Note: Means in the same column with the same lower case (^a^) indicate that the statistical differences are not significant (*p* < 0.05) by using Duncan’s test.

**Table 2:** Main chemical compositions of raw and pretreated solid digestates from straw and cattle and chicken manures.

Solid Digestate		Cellulose (%)	Hemicellulose (%)	Lignin (%)	Ash (%)	Humic Acid (%)
Straw	Raw	25.63 ± 1.23	13.42 ± 1.03	16.00 ± 1.69	25.46 ± 2.17	4.37 ± 0.12
	Pretreated	37.66 ± 1.28	11.19 ± 0.93	9.09 ± 0.71	14.23 ± 1.21	2.82 ± 0.10
Cattle manure	Raw	16.39 ± 1.20	24.22 ± 0.37	15.40 ± 1.01	8.12 ± 0.23	2.26 ± 0.13
	Pretreated	26.86 ± 1.93	19.74 ± 0.91	11.03 ± 0.09	6.23 ± 0.11	0.93 ± 0.03
Chicken manure	Raw	14.32 ± 0.01	19.26 ± 0.93	9.07 ± 0.06	4.73 ± 0.01	0.55 ± 0.001
	Pretreated	15.05 ± 0.02	12.20 ± 1.11	7.38 ± 0.10	3.23 ± 0.01	0.53 ± 0.001

**Table 3:** The crystallinity; tensile mechanical properties; water vapor permeability (WVP); thermal stability; transparency and gas transmission rate (GTR) of the composite biofilms (SA5, SA10, SA15, CT5, CT10, CT15, CK5, CK10 and CK15) and PSF.

Film	Crystallinity (%)	Mechanical Properties			WVP (10^−8^ gm/m^2^hPa)	Thermal Properties (°C)		Transparency (%)			GTR (10^−6^ m^3^/m^2^hPa)	
		Strength (MPa)	Elongation (%)	Elastic Modulus (MPa)		*T_g_*	*T_m_*	UV-B	UV-A	Vis Region	N_2_O	CH_4_
SA5	24.03 ± 0.28 ^g^	6.66 ± 0.04 ^d,e^	9.05 ± 0.36 ^a^	1567.63 ± 59.93 ^e^	3.49 ± 0.03 ^b^	67.06 ± 1.97 ^f,g^	179.57 ± 2.00 ^d^	0.91 ± 0.07 ^a^	6.54 ± 0.34 ^b^	38.89 ± 0.47 ^c^	2.68 ± 0.02 ^b^	0.98 ± 0.01 ^b^
SA10	22.36 ± 0.52 ^f^	7.49 ± 0.52 ^f^	12.67 ± 0.17 ^c,d^	1179.90 ± 9.57 ^d^	4.09 ± 0.01 ^f^	60.44 ± 0.50 ^d^	151.46 ± 2.34 ^c^	1.12 ± 0.06 ^a^	7.83 ± 0.47 ^c^	52.81 ± 0.32 ^g^	3.20 ± 0.01 ^f^	1.17 ± 0.01 ^f^
SA15	17.47 ± 0.19 ^c^	6.33 ± 0.30 ^d^	14.06 ± 0.01 ^d,e^	923.11 ± 15.88 ^c^	3.43 ± 0.01 ^b^	55.64 ± 0.86 ^b^	140.12 ± 1.65 ^b^	5.80 ± 0.29 ^c^	19.97 ± 0.71 ^e^	59.26 ± 0.22 ^i^	2.85 ± 0.01 ^b^	1.04 ± 0.01 ^b^
CT5	22.22 ± 0.55 ^f^	7.74 ± 0.29 ^f^	9.91 ± 0.26 ^a,b^	1562.37 ± 36.25 ^e^	3.91 ± 0.01 ^c,d^	68.39 ± 1.36 ^g^	222.86 ± 0.67 ^f^	1.09 ± 0.08 ^a^	5.54 ± 0.28 ^a^	32.91 ± 0.17 ^a^	3.25 ± 0.01 ^c,d^	1.18 ± 0.01 ^c,d^
CT10	19.37 ± 0.08 ^d^	5.40 ± 0.14 ^c^	11.64 ± 0.07 ^c^	912.23 ± 2.03 ^c^	3.82 ± 0.01 ^c^	63.56 ± 0.81 ^e^	218.71 ± 1.38 ^e^	3.94 ± 0.25 ^b^	14.02 ± 0.53 ^d^	52.08 ± 0.58 ^f^	3.18 ± 0.01 ^c^	1.15 ± 0.01 ^c^
CT15	17.27 ± 0.31 ^c^	4.43 ± 0.04 ^b^	14.63 ± 0.74 ^e^	687.67 ± 31.12 ^b^	3.19 ± 0.05 ^a^	57.49 ± 1.97 ^b,c^	152.81 ± 1.04 ^c^	17.26 ± 0.51 ^e^	30.39 ± 0.53 ^h^	57.07 ± 0.40 ^h^	2.41 ± 0.04 ^a^	0.88 ± 0.02 ^a^
CK5	20.97 ± 0.38 ^e^	6.97 ± 0.21 ^e^	9.35 ± 0.13 ^a^	1314.10 ± 3.63 ^d^	4.09 ± 0.01 ^f^	66.09 ± 0.26 ^f^	232.63 ± 1.39 ^g^	1.04 ± 0.06 ^a^	7.51 ± 0.44 ^c^	33.67 ± 0.15 ^b^	2.98 ± 0.01 ^f^	1.08 ± 0.01 ^f^
CK10	18.13 ± 0.73 ^c^	4.13 ± 0.23 ^b^	11.42 ± 0.15 ^b,c^	926.70 ± 91.74 ^c^	4.03 ± 0.01 ^e,f^	60.61 ± 0.38 ^d^	224.56 ± 0.77 ^f^	9.53 ± 0.33 ^d^	23.83 ± 0.61 ^f^	46.01 ± 0.18 ^d^	3.16 ± 0.01 ^e,f^	1.15 ± 0.01 ^e,f^
CK15	16.31 ± 0.04 ^b^	6.13 ± 0.19 ^d^	18.17 ± 1.38 ^f^	707.08 ± 94.44 ^b^	3.93 ± 0.01 ^d,e^	58.70 ± 0.17 ^c,d^	219.79 ± 1.67 ^e^	13.76 ± 0.39 ^e^	28.44 ± 0.61 ^g^	48.79 ± 0.25 ^e^	2.91 ± 0.01 ^d,c^	1.06 ± 0.01 ^d,c^
PSF	15.16 ± 0.10 ^a^	3.04 ± 0.02 ^a^	21.34 ± 0.12 ^e^	326.26 ± 0.01 ^a^	4.73 ± 0.09 ^g^	42.99 ± 1.00 ^a^	101.00 ± 0.01 ^a^	56.02 ± 0.69 ^f^	67.49 ± 0.20 ^i^	77.91 ± 0.24 ^j^	3.64 ± 0.07 ^g^	1.32 ± 0.03 ^g^

Note: Means in the same column with different lower cases (^a–j^) are significantly different (*p* < 0.05) by using Duncan’s test. *T_g_* and *T_m_*: glass transition temperature and degradation temperature, respectively.

## Data Availability

The data used to support the findings of this study are available from the corresponding author upon request.

## Sample Availability

Samples are available from the corresponding author upon request.

## Nomenclature

*T*_*g*_	Glass transition temperature, °C
*T*_*m*_	Degradation temperature, °C
GHC	Greenhouse gas
SEM	Scanning electron microscopy
*X*_*C*_	Relative crystalline degree, dimensionless
*I*_*C*_	Intensity of crystalline region, dimensionless
*I*_*A*_	Intensity of amorphous region, dimensionless
FTIR	Fourier-transform infrared spectroscopy
*A*_*b*_	Absorbance, dimensionless
*T*_*y*_	Transmittance (%)
RH	Relative humidity, %
WVTR	Water vapor transmission rate, g/m^2^h
WVP	Water vapor permeability, gm/m^2^hPa
*M*	Weight gain of beaker, g
*t*	Time when the change is less than 0.01 g, h
*A*	Effective area of film, m^2^
*T*	Thickness of film, m
*P*	Saturation vapor pressure of water, Pa
*R*_1_	Relative humidity value in beaker
*R*_2_	Relative humidity value in desiccator
GTR	Gas transmission rate, 10^−6^ m^3^/m^2^hPa
N_2_O	Nitrous oxide
CH_4_	Methane
XRD	X-ray diffraction
DSC	Differential scanning calorimeter
